# Effect of catheter ablation on clinical outcomes in patients with atrial fibrillation and significant functional mitral regurgitation

**DOI:** 10.1186/s12872-021-02397-5

**Published:** 2021-12-07

**Authors:** Jin-Tao Wu, Dan-Qing Zhao, Fu-Tao Zhang, Xiao-Jie Liu, Juan Hu, Lei-Ming Zhang, Xian-Wei Fan, Hai-Tao Yang, Li-Jie Yan, Jing-Jing Liu, Shan-Ling Wang

**Affiliations:** Department of Cardiology, Henan University People’s Hospital, Henan Provincial People’s Hospital, Zhengzhou, China

**Keywords:** Atrial fibrillation, Catheter ablation, Functional mitral regurgitation, Drug therapy

## Abstract

**Background:**

In patients with atrial fibrillation (AF) and functional mitral regurgitation (MR), catheter ablation reduces the severity of MR and improves cardiac remodeling. However, its effects on prognosis are uncertain.

**Methods:**

This retrospective study included 151 consecutive patients with AF and functional MR, 82 (54.3%) of whom were treated by catheter ablation (Ablation group) and 69 (45.7%) with drug therapy without ablation (Non-ablation group). Forty-three pairs of these patients were propensity matched on the basis of age, CHA_2_DS_2_-VASc scores, and left ventricular ejection fraction. The primary outcome evaluated was severity of MR, cardiac remodeling and the combined incidence of subsequent heart failure-related hospitalization and strokes/transient ischemic attacks.

**Results:**

Patients in the Ablation group showed a significant decrease in the severity of MR (*p* < 0.001), a significant decrease in the left atrial diameter (*p* = 0.010), and significant improvement in the left ventricular ejection fraction (*p* = 0.015). However, patients in the Non-ablation group showed only a significant decrease in the severity of MR (*p* = 0.004). The annual incidence of the studied events was 4.9% in the Ablation group and 16.7% in the Non-ablation group, the incidence being significantly lower in the ablation than Non-ablation group (*p* = 0.026) according to Kaplan–Meier curve analyses. According to multivariate Cox regression analysis, catheter ablation therapy (hazard ratio [HR] 0.27, 95% confidence interval [CI] 0.09–0.84; *p* = 0.024) and heart failure at baseline (HR 3.84, 95% CI 1.07–13.74; *p* = 0.038) were independent predictors of the incidence of the studied events.

**Conclusions:**

Among patients with AF and functional MR, catheter ablation was associated with a significantly lower combined risk of heart failure-related hospitalization and stroke than in a matched cohort of patients receiving drug therapy alone.

## Background

Atrial fibrillation (AF) and functional mitral regurgitation (MR) frequently coexist and exacerbate each other [1, 2]. The combination of these two conditions increases affected patients’ cardiovascular mortality and hospitalization rates [3], indicating an urgent need for developing effective therapies for these patients. Simultaneous treatment of AF and MR is ideal. However, surgical therapy is not recommended for treatment of isolated refractory AF [4]. Additionally, many patients with functional MR are not referred for mitral valve surgery because of a high surgical risk or comorbidities and a lack of proven mortality benefit [5, 6]. Catheter ablation is currently considered the treatment of choice for symptomatic drug-refractory AF [4]. Catheter ablation of AF in patients with functional MR is reportedly very effective in reducing the severity of MR [2, 7] and improving cardiac remodeling [7]. However, to the best of our knowledge, no study has assessed the effect of catheter ablation of AF on the prognosis of this particular subset of patients. Therefore, the present study was performed to compare clinical outcomes among patients with AF who had developed functional MR and underwent catheter ablation versus those treated with medical therapy only and to evaluate the effect of catheter ablation of AF on the prognosis of these patients.

## Methods

### Patients

Consecutive patients with AF who had been hospitalized in Henan Provincial People’s Hospital for diagnosis and treatment between January 2018 and December 2019 were retrospectively reviewed. Reports of transthoracic echocardiograms that had been performed before deciding on a treatment strategy were screened. MR was defined as functional if leaflets showed normal morphology but did not properly coapt because of either left ventricular (LV) or left atrial (LA) dilatation [8]. Functional MR was classified as either absent or as mild, moderate, or severe MR [8]. Moderate and severe MR were considered significant in the present study. The inclusion criteria were (a) age of < 80 years, (b) moderate or severe MR, (c) LA diameter of < 55 mm, and (d) LV ejection fraction (LVEF) of ≥ 35%. The exclusion criteria were (a) previous AF ablations; (b) previous cardiac surgery or congenital heart disease; and (c) primary MR (mitral valve prolapse, rheumatic disease, endocarditis, previous papillary muscle rupture, or abnormalities in mitral valve leaflets or chordae). We divided patients into two groups according to catheter ablation therapy status, the Ablation group comprising patients who had undergone AF ablation and the Non-ablation group comprising patients who had undergone only conventional drug therapy and not AF ablation. Whether or not to perform AF ablation was decided in accordance with the patient’s preference and the operator's discretion. Propensity-matching techniques were then used to select two subsets of patients that were as similar as possible for outcomes analysis. The study complied with the Declaration of Helsinki and the study protocol was approved by the local Institutional Review Board.

### Catheter Ablation strategy

Patients in the Ablation group had undergone AF ablation. After obtaining written informed consent, ablation was performed with the patient in a post-absorptive state under conscious sedation. Intravenous heparin was administered during the procedure, doses being adjusted to achieve an activation clotting time of > 300 ms.

The CARTO 3-dimensional electro-anatomical mapping system (Biosense Webster, Diamond Bar, CA, USA) was used in the majority of procedures. Ablation techniques varied according to the operator’s discretion, anatomical features, and type of AF. Techniques included ipsilateral pulmonary vein isolation (PVI) and modification of the atrial substrate by mapping and ablation of complex fractionated atrial electrograms (CFAEs), the cavotricuspid isthmus, and/or additional LA linear ablation, such as of the roof line, posterior box lesion, or mitral valve line from the annulus to the inferior pulmonary vein (PV) [7]. During PVI, we used the technique of circumferential PV ablation guided by three-dimensional LA mapping, which has previously been described in detail [9]. Briefly, the LA was explored via a trans-septal approach. The LA geometry was reconstructed with a 3.5-mm tip Thermocool SmartTouch catheter (Biosense Webster) in a CARTO 3-dimensional electro-anatomical mapping system. Continuous irrigated radiofrequency ablation was performed along each PV antrum to encircle the ipsilateral PVs. Ablation was delivered point by point with Thermocool SmartTouch catheters in power-controlled mode at 35 W. The target ablation index was 380 to 400 for the LA posterior wall and 500 elsewhere. The target temperature was 43 °C, and the infusion rate was 17 mL/min. The procedural endpoints were completeness of continuous circular lesions and electrical isolation of all PVs identified by a decapolar circumferential mapping catheter (Lasso; Biosense Webster).

### Guideline-directed medical therapy

Patients in the Non-ablation group had undergone conventional medical treatment but not ablation during the follow-up period. Their attending physicians had selected their medications in accordance with published guidelines [5, 10]. Medications included rate control agents (B-blockers, non-dihydropyridine calcium channel blockers, and digoxin), antiarrhythmic drugs, and anticoagulants.

### Patient follow-up

The primary clinical outcomes evaluated were the combined incidence of adverse events, including heart failure-related hospitalization or stroke/transient ischemic attack (TIA). All study patients were followed up until one of these events had occurred or until December 2020, whichever came first. All of them were contacted by telephone every 6 months and the participants or their relatives or carers interviewed regarding occurrence of any interval target clinical events. For all reported events, medical records were retrieved and reviewed. Heart failure-related hospitalization was defined as hospital admission for advanced symptoms of heart failure. Stroke was defined as a permanent neurological disability or impairment caused by various types of hemorrhagic and ischemic strokes [11]. TIA was diagnosed in accordance with the World Health Organization criteria [12], namely rapidly developing clinical evidence of focal or global disturbance of cerebral function, lasting less than 24 h, and with no apparent non-vascular cause. Subsequent echocardiographic follow-up was performed in our institution or in the patient’s home institution. Recurrence of AF was defined as occurrence of confirmed atrial tachyarrhythmia lasting longer than 30 s (documented by ECG or Holter recordings) more than 3 months after catheter ablation [10].

### Statistical analysis

All analyses were performed using SPSS software version 24.0 (IBM Corp., Armonk, NY, USA). Continuous variables are presented as mean ± standard deviation, and discrete variables are presented as percentages. Comparisons between variables were made using unpaired independent-samples *t*-tests for continuous variables, and the Mann–Whitney U-test for discrete variables. Categorical variables are presented as numbers and percentages of the group total and were compared using the χ^2^ test or Fisher’s exact test as appropriate. Patient characteristics were first compared among the entire group of eligible patients. They were then compared between the propensity-matched subsamples to ensure that the matching process had resulted in well-balanced groups. A Kaplan–Meier estimation with a log-rank test was performed for unadjusted analysis of the impact of AF ablation on the primary clinical outcomes in the propensity-matched Ablation and Non-ablation groups. Univariable Cox proportional hazards regression analysis was used to identify clinical and other patient variables associated with clinical outcomes during follow-up. Variables that showed *P* < 0.10 in univariable analysis were included in the multivariable models (forward likelihood ratio). All probability values were two-sided. *P* < 0.05 was considered to denote statistical significance.

## Results

### Study cohort

Between January 2018 and December 2019, 1,263 patients with AF were screened, with 156 of them found to have significant functional MR and enrolled in the present study. Of these 156 patients, 151 completed follow-up (96.8%) and comprised the study cohort. AF ablation had been performed on 54.3% (82/151) of these patients, the remaining 45.7% (69/151) having undergone only conventional drug therapy and not AF ablation. From this sample, a propensity-matching algorithm produced 43 pairs of patients with similar propensities based on age, CHA_2_DS_2_-VASc, and LVEF. Patient characteristics for both the entire initial cohort of patients who met the selection criteria and completed follow-up and for the final propensity-matched samples are summarized in Table 1. Before matching, several differences between the ablation and non-ablation cohorts were noted. Specifically, the patients who had undergone ablation were younger, had lower CHA_2_DS_2_-VASc scores, smaller LA diameters, smaller LV end-systolic dimensions, and higher LVEF than did those who had not undergone AF ablation. The prevalences of stroke/TIA, coronary artery disease, chronic kidney disease, and antiarrhythmic drug use were all higher in ablation patients than in those who did not undergo ablation. After matching, the patient characteristics appeared well balanced between groups, the only statistically significant difference between them being the LA diameter.

**Table 1:** Patient characteristics before and after propensity matching

	Before matching			After matching		
	Ablation (n = 82)	Nonablation (n = 69)	*P* value	Ablation (n = 43)	Nonablation (n = 43)	*P* value
Age, years	65.2 ± 8.5	70.1 ± 7.6	< 0.001	68.3 ± 7.6	68.0 ± 8.4	0.851
Male, n (%)	44 (53.7%)	36 (52.2%)	0.856	20 (46.5%)	25 (58.1%)	0.280
AF duration, months (median, IQR)	12.0 (2–48)	24.0 (4–36)	0.540	12.0 (3–48)	24.0 (1–36)	0.447
Paroxysmal AF, n (%)	22 (26.8%)	14 (20.3%)	0.348	13 (30.2%)	8 (18.6%)	0.209
Persistent AF, n (%)	60 (73.2%)	55 (79.7%)		30 (69.8%)	35 (81.4%)	
CHA_2_DS_2_-VASc score	2.4 ± 1.6	3.5 ± 1.8	< 0.001	3.1 ± 1.6	2.9 ± 1.8	0.564
Diabetes mellitus, n (%)	13 (15.9%)	13 (18.8%)	0.628	9 (20.9%)	9 (20.9%)	1.000
Hypertension, n (%)	46 (56.1%)	36 (52.2%)	0.630	24 (55.8%)	20 (46.5%)	0.388
Previous stroke or TIA, n (%)	10 (12.2%)	19 (27.5%)	0.017	9 (20.9%)	11 (25.6%)	0.610
Heart failure, n (%)	5 (6.1%)	4 (5.8%)	1.000	4 (9.3%)	2 (4.7%)	0.676
Coronary artery disease, n (%)	19 (23.2%)	31 (44.9%)	0.005	16 (37.2%)	12 (27.9%)	0.357
Chronic kidney disease, n (%)	1 (1.2%)	7 (10.1%)	0.024	1 (2.3%)	4 (9.3%)	0.360
Echocardiogram						
LVEF, %	58.4 ± 8.1	54.1 ± 9.4	0.003	56.9 ± 9.1	55.9 ± 9.3	0.599
LA diameter, mm	43.8 ± 4.8	46.4 ± 4.7	0.001	43.1 ± 4.7	46.5 ± 4.8	0.001
LVESD, mm	33.4 ± 5.8	36.3 ± 7.6	0.010	34.0 ± 6.9	35.2 ± 7.2	0.409
LVEDD, mm	49.1 ± 5.4	50.7 ± 7.6	0.138	49.1 ± 5.6	50.3 ± 7.3	0.400
Medication use						
Anticoagulants, n (%)	60 (73.2%)	54 (78.3%)	0.469	26 (60.5%)	29 (67.4%)	0.500
β-blockers, n (%)	57 (69.5%)	46 (66.7%)	0.708	32 (74.4%)	27 (62.8%)	0.245
Antiarrhythmics (Class I or III), n (%)	34 (41.5%)	12 (17.4%)	0.001	8 (18.6%)	7 (16.3%)	0.776
Calcium channel blockers, n (%)	13 (15.9%)	13 (18.8%)	0.628	7 (16.3%)	7 (16.3%)	1.000
Digoxin, n (%)	16 (19.5%)	21 (30.4%)	0.120	11 (25.6%)	9 (20.9%)	0.610
Follow-up, months	22.4 ± 8.1	19.6 ± 9.2	0.051	22.9 ± 7.9	20.1 ± 9.6	0.151

AF, atrial fibrillation; IQR, interquartile range; TIA, transient ischemic attacks; LVEF, left ventricular ejection fraction; LA, left atrial; LVEDD, left ventricular end-diastolic dimensions; LVESD, left ventricular end-systolic dimensions

### AF Ablation efficacy outcome

PVI was achieved in all patients in the Ablation group during the initial procedure. A cavotricuspid isthmus line was created in 27 (62.8%) patients and CFAEs were also ablated during the first procedure in 18 (41.9%) patients. Thirty (69.8%) patients had additional LA linear ablation. There were no procedural complications in the Ablation group. During the mean follow-up period of 16.7 ± 10.2 months (range 3–35 months), 20 patients (46.5%) developed recurrences of AF. Only two patients underwent repeat procedures over the entire follow-up period.

### Follow-up echocardiography

**Fig. 1:**
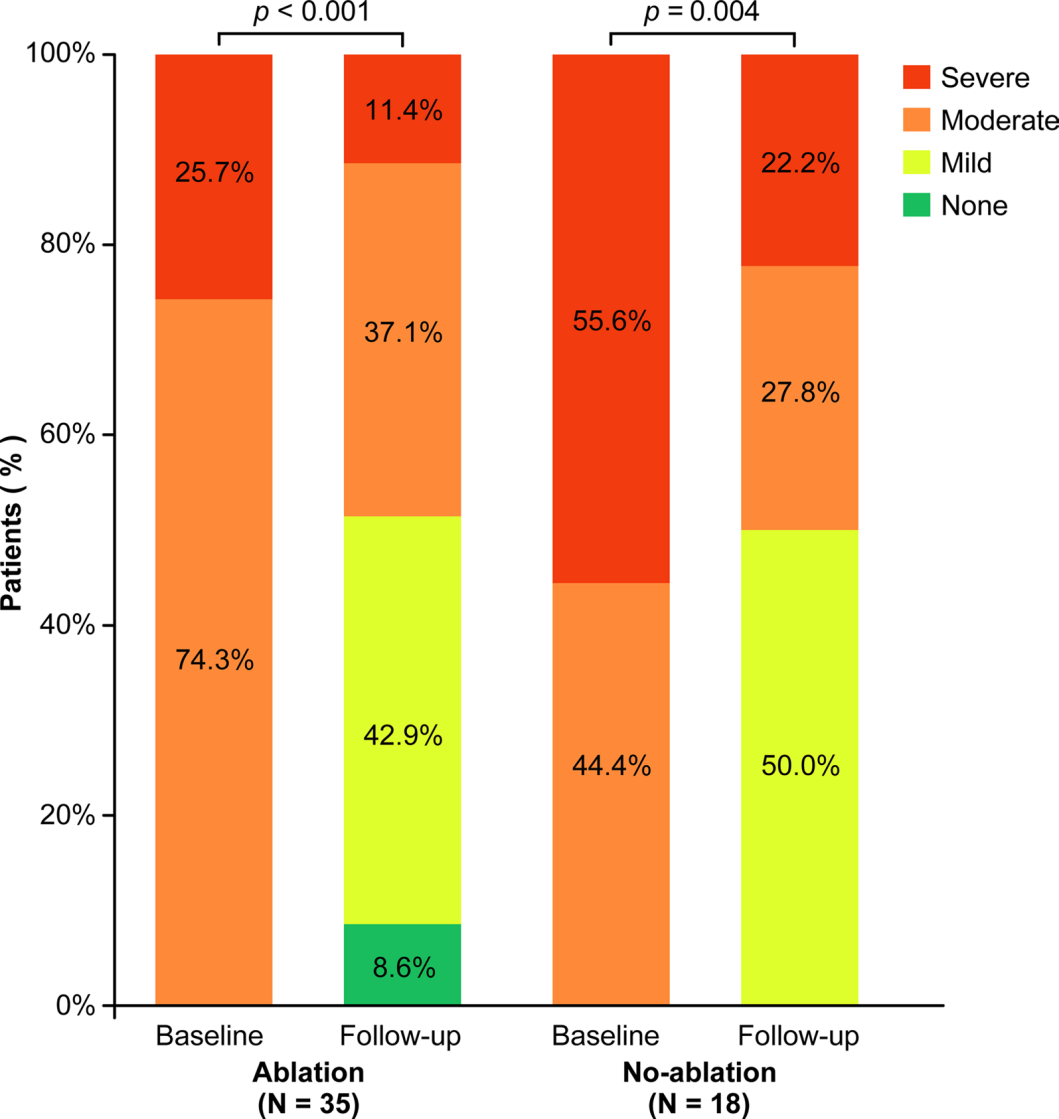
Severity of mitral regurgitation at baseline and during follow-up according to whether catheter ablation therapy was performed

**Fig. 2:**
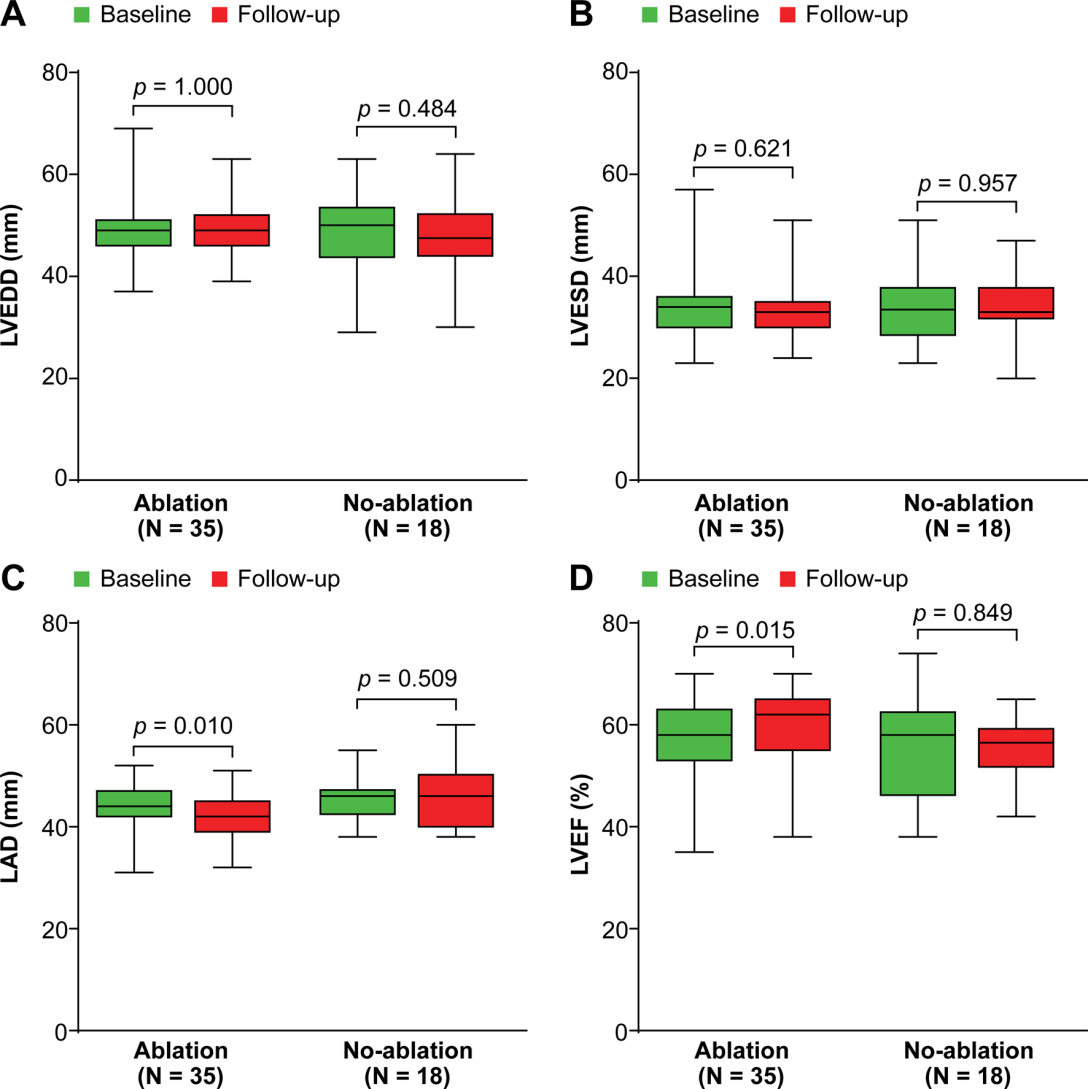
Change in **A**, **B** left ventricular dimensions, **C** left atrial diameter, and **D** left ventricular ejection fraction from baseline to follow-up. LVEDD, left ventricular end-diastolic dimension; LVESD, left ventricular end-systolic dimension; LAD, left atrial diameter; LVEF, left ventricular ejection fraction

Follow-up echocardiograms were available in 53 of the 86 patients at a mean of 8.6 ± 6.6 months after the initial procedure. Of these 53 patients, 35 were in the Ablation group and 18 were in the Non-ablation group. Patients in the Ablation group showed a significant decrease in the severity of MR (*p* < 0.001), a significant decrease in the LA diameter (*p* = 0.010), and significant improvement in the LVEF (*p* = 0.015) compared with baseline. However, they showed no significant difference in the LV end-diastolic dimension (*p* = 0.621) or LV end-systolic dimension (*p* = 1.000) compared with baseline (Figs. 1, 2). Patients in the Non-ablation group showed a significant decrease in the severity of MR (*p* = 0.004), but there were no significant differences in the LV end-diastolic dimension (*p* = 0.957), LV end-systolic dimension (*p* = 0.484), LA diameter (*p* = 0.509), or LVEF (*p* = 0.849) (Figs. 1, 2).

### Comparison of clinical outcomes

**Fig. 3:**
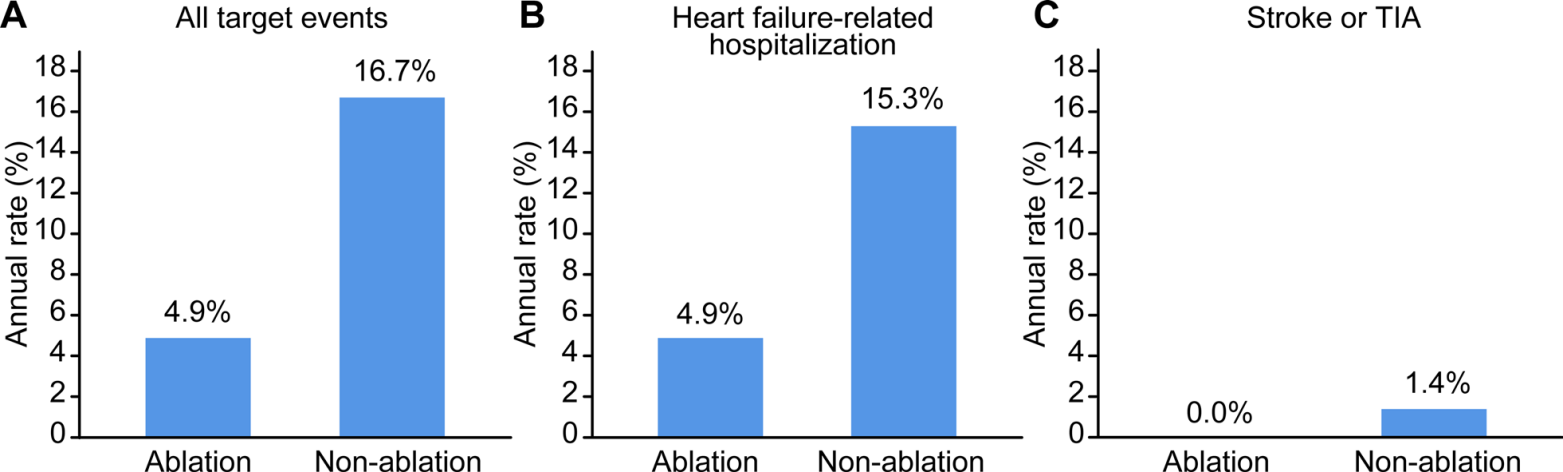
Annual rates of the indicated events according to catheter ablation therapy status. **A** All target events. **B** Heart failure-related hospitalizations. **C** Stroke or transient ischemic attacks (TIA)

**Fig. 4:**
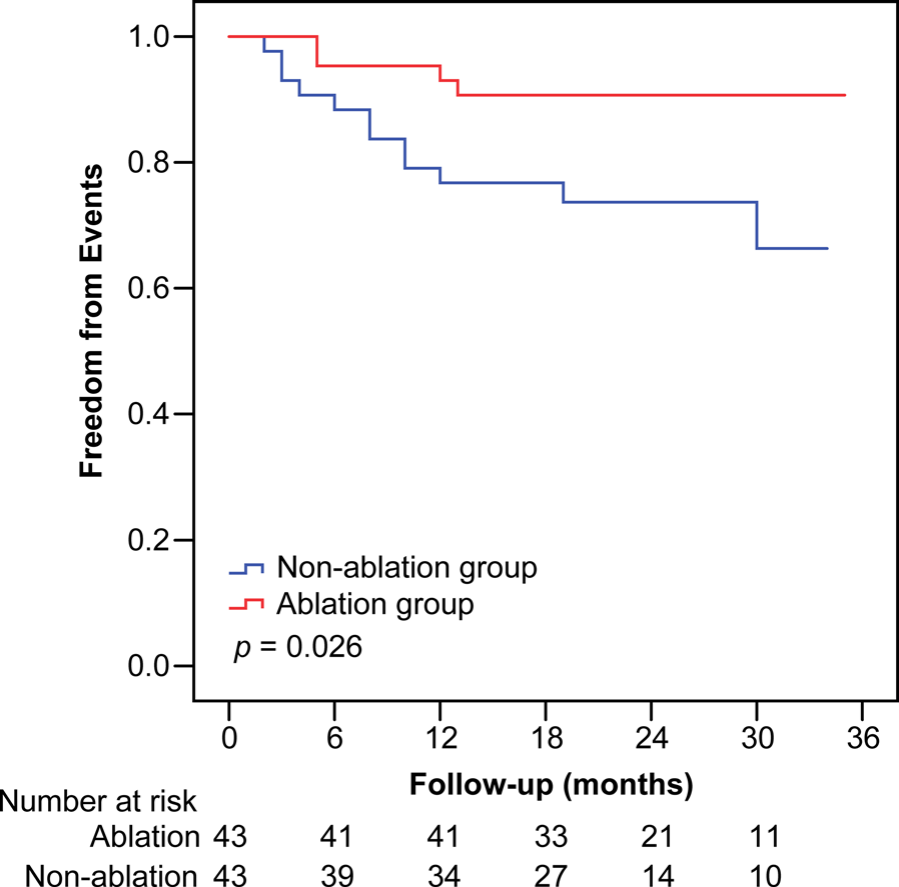
Kaplan–Meier curves of the combined incidence of target clinical events according to catheter ablation therapy status. The incidence of combined clinical events is significantly lower in the Ablation than Non-ablation group (9.3% vs. 27.9%, *P* = 0.026 by log-rank test)

The mean follow-up duration in the Ablation and Non-ablation group was 22.6 ± 8.1 and 20.1 ± 9.6 months, respectively. During the entire follow-up period, heart failure-related hospitalization was required by four patients (9.3%) in the Ablation group. There were no stroke or TIA events in this group. Thus, only four patients (9.3%) in the Ablation group had a target clinical event, namely heart failure-related hospitalization or new-onset stroke. In contrast, 11 patients (25.6%) in the Non-ablation group had heart failure-related hospitalization and one had a stroke event. Thus, target clinical events occurred in 12 patients (27.9%) in the Non-ablation group. The overall annual rate of heart failure-related hospitalization was 4.9% in the Ablation group and 15.3% in the Non-ablation group (Fig. 3B). The overall annual rate of stroke or TIA was 0% in the Ablation group and 1.4% in the Non-ablation group (Fig. 3C). The combined overall annual rate of target clinical events was 4.9% in the Ablation group and 16.7% in the Non-ablation group (Fig. 3A). Kaplan–Meier curves for the combined incidence of target clinical events showed that event-free rates were significantly higher in the Ablation than the Non-ablation group (*p* = 0.026) (Fig. 4).

### Predictors of the incidence of clinical events

Predictors of the incidence of target clinical events in the entire patient cohort were determined using a Cox proportional hazards model. Univariable Cox analysis showed that catheter ablation therapy (hazard ratio [HR] 0.30, 95% confidence interval [CI] 0.10–0.94; *p* = 0.038) was associated with the occurrence of target clinical events (Table 2). According to multivariate analysis, catheter ablation therapy (HR 0.27, 95% CI 0.09–0.84; *p* = 0.024) and heart failure at baseline (HR 3.84, 95% CI 1.07–13.72; *p* = 0.039) were independent predictors of the incidence of target clinical events (Table 2).

**Table 2:** Univariate and multivariate predictors of target clinical events

	Univariate Cox regression			Multivariate Cox regression		
	HR	95% CI	*P* value	HR	95% CI	*P* value
Age	1.03	0.97–1.10	0.303			
Female	1.16	0.43–3.13	0.763			
Body mass index	1.08	0.94–1.24	0.288			
AF duration	1.01	1.00–1.02	0.172			
Persistent AF	2.37	0.54–10.45	0.254			
CHA_2_DS_2_-VASc score	1.28	0.97–1.68	0.085			
Diabetes mellitus	0.83	0.24–2.91	0.767			
Hypertension	1.30	0.48–3.49	0.609			
Previous stroke or TIA	2.16	0.78–5.94	0.137			
Heart failure	3.01	0.86–10.58	0.086	3.84	1.07–13.74	0.038
Coronary artery disease	1.22	0.44–3.35	0.702			
Chronic kidney disease	2.65	0.60–11.78	0.199			
LVEF	0.99	0.94–1.04	0.718			
LA diameter	1.04	0.94–1.15	0.420			
LVESD	1.02	0.95–1.09	0.621			
LVEDD	1.02	0.95–1.10	0.606			
Anticoagulants	2.57	0.73–9.01	0.141			
β-blockers	1.92	0.54–6.77	0.313			
Antiarrhythmics (Class I or III)	1.06	0.30–3.75	0.923			
Calcium channel blockers	0.69	0.16–3.04	0.624			
Digoxin	1.03	0.33–3.18	0.966			
Catheter ablation therapy	0.30	0.10–0.93	0.037	0.27	0.09–0.84	0.024

AF, atrial fibrillation; TIA, transient ischemic attacks; LVEF, left ventricular ejection fraction; LA, left atrial; LVEDD, left ventricular end-diastolic dimensions; LVESD, left ventricular end-systolic dimensions; HR, hazard ratio; CI, confidence interval

## Discussion

The main findings in this study were as follows: (1) in patients with AF and significant functional MR, catheter ablation is associated with a significantly lower risk of target clinical events (heart failure-related hospitalization, stroke, and TIA) than is conventional drug therapy. (2) In these patients, catheter ablation reduces the severity of MR and improves cardiac remodeling.

A previous study evaluating the prognostic significance of residual functional MR in hospitalized patients with chronic AF and heart failure but a preserved LVEF showed that after optimized drug therapies, the mean MR grade at discharge was significantly lower than that during hospitalization [13]. These findings suggest that conventional drug therapy alone may significantly reduce the severity of MR in patients with AF and functional MR, which is supported by our finding that patients in the Non-ablation group showed a significant decrease in the severity of MR during the follow-up period compared with baseline. In addition, several studies have examined the efficacy of catheter ablation of AF in patients with MR. A study retrospectively compared 53 patients with significant functional MR and normal LV systolic function (LVEF ≥ 50%) with a matched AF cohort with trivial and/or mild MR during first AF ablation [2]. This previous study showed that successful ablations were associated with a significant reduction in severity of MR and LA size. In a recent study investigating the outcomes of catheter ablation of AF in a subgroup of patients presenting with functional MR and LV systolic dysfunction, we also found associations between freedom from recurrent atrial tachyarrhythmia after ablation and a reduction in severity of MR and with positive LA and LV remodeling [7]. Our findings suggest that, similarly to patients with AF with functional MR and normal LV function, patients with AF with functional MR and LVSD also benefit from restoration of sinus rhythm by catheter ablation of AF. However, whether catheter ablation is more beneficial than conventional drug therapy in improving clinical outcomes in patients with AF and functional MR is unclear.

In this comparative study of catheter ablation versus medical therapy in patients with AF and functional MR, we found lower annual overall rates of target clinical events in the Ablation than in the Non-ablation group. According to univariate and multivariate analyses, catheter ablation therapy is significantly associated with fewer subsequent target clinical events, namely heart failure-related hospitalization and strokes/TIA. Our findings suggest that the rates of clinical outcomes may be more effectively reduced by catheter ablation than by conventional drug therapy in patients with AF and functional MR. This may be explained by our finding that besides a significant reduction in the severity of MR in the two groups, patients in the Ablation group also showed a significant decrease in the LA diameter and improvement in the LVEF whereas patients in the Non-ablation group did not.

In our previous study, we showed that freedom from recurrent AF is associated with a reduction in the severity of MR and positive cardiac reverse remodeling, whereas patients with AF recurrence after AF ablation do not experience these benefits [7]. These findings suggest that maintenance of sinus rhythm after ablation is important in achieving reduction in the severity of MR and positive cardiac reverse remodeling, and may be associated with subsequent better clinical outcomes. However, AF patients with functional MR have high recurrence rates after AF ablation [14]. A previous study of 216 patients with long-standing persistent AF who underwent catheter ablation identified both MR and LA size as independent predictors of recurrence of AF [15]. In the current study, we found a 46.5% rate of recurrence of AF during a mean follow-up period of 16.7 ± 10.2 months after a single ablation procedure. Because the rates of recurrence of AF after ablation are higher with longer term follow-up, whether catheter ablation is still associated with a lower risk of clinical events than conventional drug therapy during longer term follow-up is unknown. Therefore, further studies incorporating long-term follow-up are required for these patients.

Among patients with AF and functional MR, some have normal LV systolic function, in which functional MR develops as a result of LA dilatation. This MR is known as atrial functional MR[16]. However, in present study, we did not stratify patients with atrial functional MR or functional MR secondary to LV dilatation for two reasons. First, atrial functional MR is not well understood and is still under evaluation. The definition of “atrial functional MR” is yet not widely adopted. Second, because AF can also result in LV systolic dysfunction due to loss of atrioventricular synchrony or can be a direct cause of tachycardia-induced ventricular cardiomyopathy, patients with AF and functional MR usually have LV dilatation. Additionally, LV dilatation can precede functional MR and AF because pre-existing ventricular cardiomyopathy can result in subsequent functional MR and atrial dilatation, which increase the likelihood of AF development [17, 18]. Thus, for the majority of patients with AF co-existent with LV dilatation and functional MR, the mechanism of MR usually includes both LV and LA dilatation, and it is difficult to differentiate the mechanism of MR from LV dilatation or LA dilatation.

Several limitations of our study should be considered. First, the small sample size is a major limitation of this study and may have introduced statistical bias. Further studies with larger sample sizes are needed. Second, because there were multiple differences at baseline between the Ablation and Non-ablation groups, propensity-matching techniques were used in this study. Because the sample size was small, we did not match all of the differences between the two groups. The patients’ age [19], CHA_2_DS_2_-VASc scores [20], and LVEF [21, 22] were significantly associated with the target clinical events (heart failure-related hospitalization or strokes/TIA); therefore, the patients were propensity score-matched on the basis of these three parameters. Fortunately, the patient characteristics appeared well balanced between the two groups after matching; the only statistically significant difference between them was the LA diameter. However, multivariable Cox analysis showed that the LA diameter was not an independent predictor of the target clinical events, suggesting that this bias would not have affected our conclusions. Third, for patients with persistent AF, we performed ipsilateral PVI and atrial substrate modification. However, different patients may have been treated by different techniques of atrial substrate modification according to the operator’s discretion and patients’ clinical features, and this may have introduced bias. Notably, the current guidelines contain no consensus regarding the techniques of atrial substrate modification for persistent AF [4]. Further prospective studies in which the same atrial substrate modification techniques are used may be required. Fourth, follow-up echocardiograms were available in only 53 of the 86 patients, including 81.4% (35/43) patients in the Ablation group and 41.9% (18/43) patients in the Non-ablation group. Although this may not have affected our findings in the Ablation group, the findings regarding the severity of MR and cardiac remodeling in the Non-ablation group are not convincing because follow-up echocardiograms were available only in 41.9% (18/43) of patients. However, a previous study showed that conventional drug therapy alone may significantly reduce the severity of MR in patients with AF and functional MR [13], which supports our findings in the Non-ablation group. Further studies are required to clarify our findings. Fifth, we did not assess differences in quality of life associated with long-term drug therapy or ablation between the two study groups because these measures are not routinely captured in medical records. Finally, the generalizability of our findings may be limited by the single-center, retrospective, observational approach.

## Conclusions

Among patients with AF and functional MR, catheter ablation is associated with a significantly lower risk of clinical events than is conventional drug therapy. These findings require confirmation with randomized study designs and long-term follow up.

## Data Availability

The data set supporting the results of this article are included within the article, further inquiries can be directed to the corresponding author/s.

## Abbreviations

AF: Atrial fibrillation
CFAEs: Complex fractionated atrial electrograms
CI: Confidence interval
HR: Hazard ratio
LA: Left atrial
LV: Left ventricular
LVEF: LV ejection fraction
MR: Mitral regurgitation
PV: Pulmonary vein
PVI: Pulmonary vein isolation
TIA: Transient ischemic attack
